# 5-Year Clinical Outcomes of Successful Recanalisation for Coronary Chronic Total Occlusions in Patients With or Without Type 2 Diabetes Mellitus

**DOI:** 10.3389/fcvm.2021.691641

**Published:** 2021-08-13

**Authors:** Peizhi Wang, Deshan Yuan, Sida Jia, Pei Zhu, Ce Zhang, Yue Liu, Tianyu Li, Lin Jiang, Ying Song, Jingjing Xu, Xiaofang Tang, Xueyan Zhao, Bo Xu, Yuejin Yang, Jinqing Yuan, Runlin Gao

**Affiliations:** Department of Cardiology, Center for Coronary Heart Disease, Fuwai Hospital, National Center for Cardiovascular Diseases, Chinese Academy of Medical Sciences and Peking Union Medical College, Beijing, China

**Keywords:** chronic total occlusion, percutaneous coronary intervention, diabetes mellitus, prognosis, successful revascularization

## Abstract

**Background:** Despite substantial improvement in chronic total occlusions (CTO) revascularization technique, the long-term clinical outcomes in diabetic patients with revascularized CTO remain controversial. Our study aimed to investigate the 5-year cardiovascular survival for patients with or without type 2 diabetes mellitus (DM) who underwent successful percutaneous coronary intervention (PCI) for CTO.

**Methods:** Data of the current analysis derived from a large single-center, prospective and observational cohort study, including 10,724 patients who underwent PCI in 2013 at Fuwai Hospital. Baseline, angiographic and follow-up data were collected. The primary endpoint was major adverse cardiac and cerebrovascular events (MACCE), which consisted of death, recurrent myocardial infarction (MI), stroke and target vessel revascularization (TVR). The secondary endpoint was all-cause mortality. Cox regression analysis and propensity-score matching was performed to balance the baseline confounders.

**Results:** A total of 719 consecutive patients with ≥1 successful CTO-PCI were stratified into diabetic (*n* = 316, 43.9%) and non-diabetic (*n* = 403, 56.1%) group. During a median follow-up of 5 years, the risk of MACCE (adjusted hazard ratio [HR] 1.47, 95% confidence interval [CI] 1.08–2.00, *P* = 0.013) was significantly higher in the diabetic group than in the non-diabetic group, whereas the adjusted risk of all-cause mortality (HR 2.37, 95% CI 0.94–5.98, *P* = 0.068) was similar. In the propensity score matched population, there were no significant differences in the risk of MACCE (HR 1.27, 95% CI 0.92–1.75, *P* = 0.155) and all-cause mortality (HR 2.56, 95% CI 0.91–7.24, *P* = 0.076) between groups. Subgroup analysis and stratification analysis revealed consistent effects on 5-year MACCE across various subgroups.

**Conclusions:** In patients who received successful CTO-PCI, non-diabetic patients were related to better long-term survival benefit in terms of MACCE. The risk of 5-year MACCE appeared to be similar in less-controlled and controlled diabetic patients after successful recanalization of CTO. Further randomized studies are warranted to confirm these findings.

## Introduction

Chronic total occlusion (CTO) occurs in ~15–25% of patients with coronary artery disease (CAD) undergoing diagnostic coronary angiography (1, 2). Due to the development of interventional devices and dedicated techniques, percutaneous coronary intervention (PCI) for CTO has achieved high technical success rates with a low risk for procedural complications, especially in tertiary medical centers. Current guidelines have regarded revascularization for CTO as the IIa B recommendation (3). Considerable evidence suggest that successful CTO-PCI is related to a better improvement of symptoms, quality of life, and ventricular function compared to optimal medical treatment alone and unsuccessful CTO-PCI (4–6), whereas the benefit in terms of improving patient survival was not significant (7). The beneficial effect of CTO-PCI on long-term prognosis is still controversial, especially for the special group of people with diabetes (2, 8).

Type 2 Diabetes mellitus (DM) is a well-established CAD risk equivalent and is associated with a greater atherosclerotic burden, such as multivessel disease, heavily calcified coronary lesions, diffuse and small vessel CAD (9, 10). Previous studies have reported that patients with DM have an elevated incidence of CTO (~30–40%) (11, 12). In addition, CTO patients with DM are related to longer and more technically challenging occluded lesions, with lower success rates compared with that in non-DM (13). Besides, non-DM patients were more likely to fare better after CTO-PCI for up to 3 years compared to their DM counterparts (14). However, to the best of our knowledge, no previous study has focused on longer term impact of successful recanalisation for CTO lesions in patients with vs. without DM. Therefore, we conducted a prospective, observational and real-world study to investigate 5-year clinical outcomes in type 2 diabetic and non-diabetic patients after successful CTO-PCI.

## Materials and Methods

### Study Population

**Figure 1:**
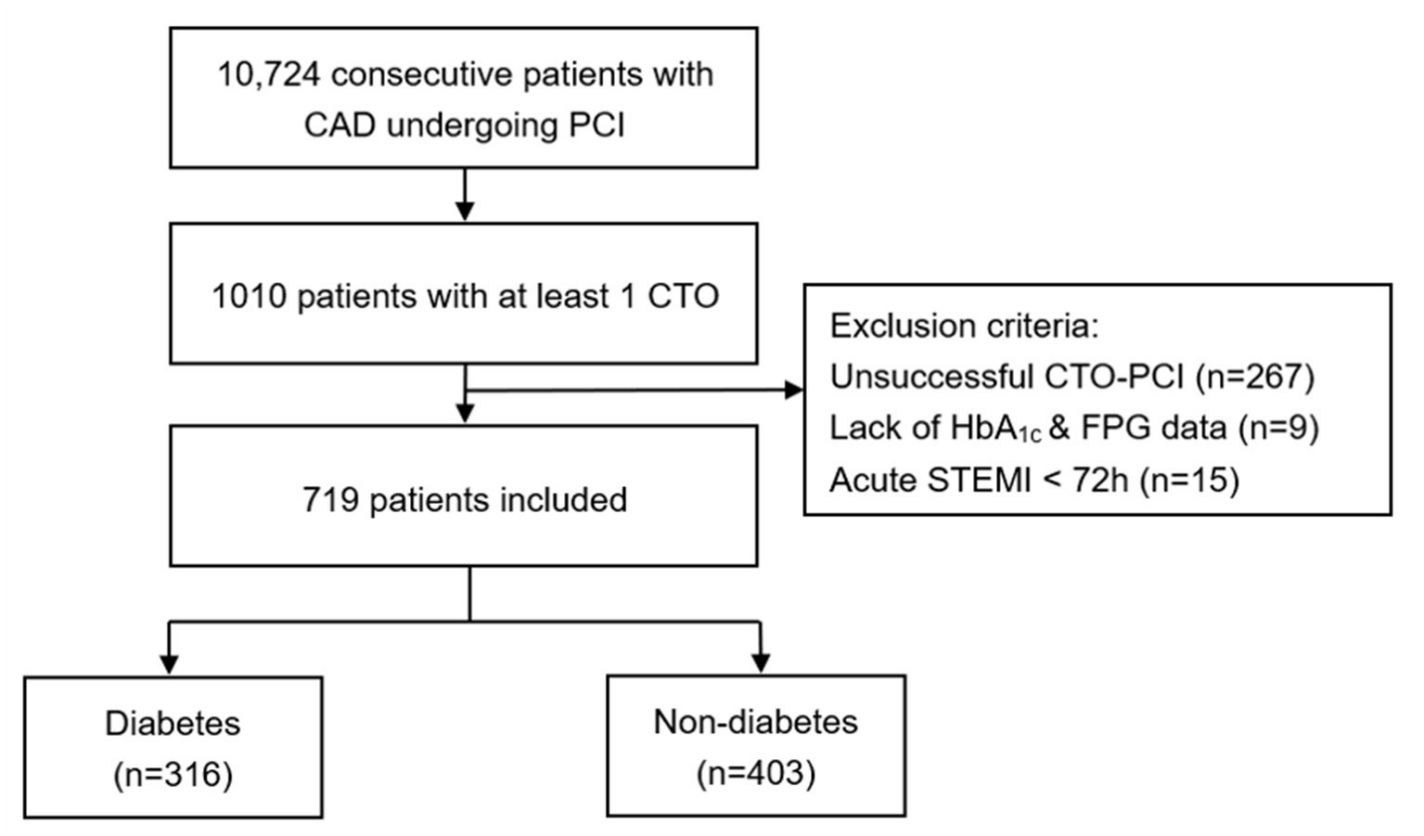
Study flow chart. CAD, coronary artery disease; PCI, percutaneous coronary intervention; CTO, chronic total occlusion; HbA1c, hemoglobin A1c; FPG, fasting plasma glucose; STEMI, ST-segment elevation myocardial infarction.

A total of 10,724 consecutive patients with CAD who underwent PCI were enrolled between January 2013 and December 2013 in Fu Wai Hospital, National Center for Cardiovascular Diseases, Beijing, China. Notably, we included 1,010 (9.42%) patients with at least 1 CTO lesion. CTO lesions were defined as complete obstruction of a native coronary artery for longer than 3 months with thrombolysis in myocardial infarction (TIMI) flow grade of 0 (15). Patients who have undergone a successful CTO-PCI were implanted with second-generation drug-eluting stents (DES) or biodegradable polymer DESs. Patients who received recanalisation treatment for CTO depended on contemporary practice guidelines, judgment from our team's experienced cardiologists and their own preference (16). Exclusion criteria included the following: (1) patients who underwent unsuccessful CTO-PCI (*n* = 267); (2) patients lacking both hemoglobin A1c (HbA1c) and fasting plasma glucose (FPG) data (*n* = 9); (3) patients who were diagnosed as acute STEMI within 72 h before admission (*n* = 15). Thus, the remaining 316 (43.9%) patients with type 2 DM and 403 (56.1%) patients without DM were enrolled for the final analysis (Figure 1). DM was defined as a FPG of at least 7.0 mmol/L, or glycated HA1c >6.5% or known diabetes, based on previous medical records of the patients and data of the therapeutic status based on the glucose-lowering therapy (17). Less-controlled DM was considered as HbA1c ≥ 7% or non-elevated FPG (18, 19). Left ventricular ejection fraction (LVEF) was measured from two-dimensional echocardiography according to modified Simpson's rule. Estimated glomerular filtration rate (eGFR) was calculated by the modified diet in renal disease equation for Chinese (20). Data of demographic, clinical and angiographic features were collected from the database and medical records retrospectively, whereas clinical endpoints during follow-up were identified prospectively. The study complied with the principles of the Declaration of Helsinki and was approved by the Institutional Ethics Committee at Fu Wai Hospital. All eligible participants gave written informed consent.

### PCI Procedures

Coronary interventions were performed according to current standard guidelines at the discretion of the operating physician (16). Before catheterization, unless on chronic P2Y12 inhibitor therapy for > 6 days, selected PCI patients received oral administration of aspirin 300 mg and clopidogrel (loading dose 300 mg) or ticagrelor (loading dose 180 mg) at least 24 h. Patients presenting as acute coronary syndrome (ACS) scheduled for PCI received the same dose of aspirin and ticagrelor or clopidogrel (loading dose 300 or 600 mg) as soon as possible. Thereafter, unfractionated heparin (100 U/kg) was administered before PCI, however, the use of glycoprotein IIb/IIIa inhibitors was at the operator's judgment. CTO-PCI was done using bilateral injections, specialized hydrophilic wires, microcatheters and retrograde approach, when available. If both antegrade and retrograde approaches failed, intravascular ultrasound (IVUS) guided wire re-entry technique would be attempted. Standard dual-antiplatelet medication was maintained for at least 12 months after PCI. The PCI procedure was considered successful if residual stenosis <30% with TIMI flow grade 3 at the end of the procedure was obtained according to visual estimation of the angiograms.

### Endpoints and Follow-Up

The primary clinical outcome was the occurrence of 5-year major adverse cardiac and cerebrovascular events (MACCE) during follow-up, a composite endpoint of death, recurrent myocardial infarction (MI), stroke and target vessel revascularization (TVR). The secondary endpoint was all-cause mortality. Death that could not be attributed to a non-cardiac etiology was considered cardiac death. MI was defined by the Third Universal Definition (21). TVR was defined as revascularization for a new lesion on the target vessel either by PCI or by surgery (22). Patients were evaluated at 1, 6, and 12 months postoperatively and annually thereafter for up to 5 years. Clinical follow-up was performed through examination of hospital records, telephone follow-up and outpatient clinical visit by research coordinators.

### Statistical Analysis

Categorical variables were compared with Chi-square test or Fisher's exact test, where applicable, and data were presented as frequencies and percentages. Continuous variables were tested using Student's *t*-test and were summarized as the mean ± standard deviation. The cumulative incidence of clinical outcomes was calculated by Kaplan–Meier analysis and compared using log-rank test. Covariates that were significant on univariate analysis (*P* < 0.10) or clinically relevant were included in multivariate models. Cox regression was used to compare adjusted hazard ratios based on age, eGFR, LVEF, prior stroke, prior PCI, prior MI, left anterior descending coronary artery (LAD) involvement and peripheral vascular disease (PVD) (Details available in Supplementary Table 1). Additionally, propensity score matching (PSM) analysis was constructed to adjust for any potential confounder in baseline characteristics between the two groups based on multivariable logistic regression model. The nearest neighbor matching algorithm was used for PSM via a 1:1 matching protocol. Exploratory subgroup analysis was carried out to assess the effect of glycemic status (DM and Non-DM) on MACCE in specific patient subsets using the same multivariable model. Similarly, stratification analysis was performed to make comparison with different groups (less-controlled DM and controlled DM, and insulin-dependent DM and non-insulin-dependent DM) on major adverse events. Cox regression analysis was also conducted to compare the DM group with non-DM group in the risk of MACCE and all-cause mortality during 2 years of follow-up. Two-tailed *P*-value of < 0.05 was considered as statistically significance. The SPSS Version 26.0 (SPSS Inc., Chicago, Illinois, USA) was used for all statistical computations.

## Results

### Baseline Patient Characteristics

The prevalence of CTO was 9.42% in the total population. Success rate of CTO-PCI was 73.6%. Among a total of 719 selected patients with at least 1 successful CTO-PCI at least in our prospective and observational cohort, 316 (43.9%) patients had DM and 69 (21.8%) were dependent on insulin (Figure 1). The baseline demographic and treatment characteristics of the patients with and without DM are shown in Table 1. Angiographic and procedural characteristics of the patients are shown in Table 2. No statistically significant differences were found in the baseline clinical and lesion characteristics between the diabetic and non-diabetic group, except for LVEF. Notably, LVEF in the two groups were all within normal range. After performing propensity score matching for the enrolled patients, 289 matched pairs of patients were created and we did not find considerable differences in the baseline clinical and lesion characteristics between the two matched groups (Tables 1, 2).

**Table 1:** Baseline clinical characteristics in the diabetes and the non-diabetes groups.

**Variables**	**Total population (** ***n*** **=** **719)**		***P*-value**	**Propensity-matched patients (** ***n*** **=** **578)**		***P*-value**
	**Diabetes (*n* = 316)**	**Non-diabetes (*n* = 403)**		**Diabetes (*n* = 289)**	**Non-diabetes (*n* = 289)**	
Age (years)	57.8 ± 10.2	56.7 ± 10.2	0.141	57.4 ± 10.2	56.6 ± 10.2	0.347
Male	261 (82.6)	342 (84.9)	0.412	240 (83.0)	243 (84.1)	0.736
Current smoking	201 (63.6)	248 (61.5)	0.570	184 (63.7)	185 (64.0)	0.931
Hypertension	209 (66.1)	245 (60.8)	0.140	186 (64.4)	180 (62.3)	0.605
Hyperlipidemia	227 (71.8)	278 (69.0)	0.406	208 (72.0)	199 (68.9)	0.412
LVEF (%), at baseline	60.2 ± 8.6	62.6 ± 6.7	<0.001	61.45 ± 7.2	62.5 ± 6.6	0.066
eGFR (mL/min)	91.3 ± 16.6	93.1 ± 13.7	0.107	92.15 ± 16.2	93.35 ± 13.7	0.340
LDL-C (mmol/L)	2.45 ± 0.9	2.51 ± 1.0	0.399	2.47 ± 0.9	2.49 ± 1.1	0.778
Fasting glucose (mmol/L)	7.11 ± 2.57	5.01 ± 1.05	<0.001	7.24 ± 2.47	5.14 ± 0.58	<0.001
HbA1c (%)	7.51 ± 1.37	5.92 ± 0.33	<0.001	7.50 ± 1.37	5.93 ± 0.34	<0.001
Prior stroke	3 (0.9)	6 (1.5)	0.738	3 (1.0)	2 (0.7)	1.000
Prior PCI	76 (24.1)	89 (22.1)	0.534	65 (22.5)	62 (21.5)	0.763
Prior MI	97 (30.7)	115 (28.5)	0.528	81 (28.0)	79 (27.3)	0.853
Prior CABG	25 (7.9)	28 (6.9)	0.624	21 (7.3)	21 (7.3)	1.000
Familial history of CAD	71 (22.5)	93 (23.1)	0.847	65 (22.5)	72 (24.9)	0.494
COPD	7 (2.2)	12 (3.0)	0.527	7 (2.4)	7 (2.4)	1.000
PVD	8 (2.5)	10 (2.5)	0.966	3 (1.0)	7 (2.4)	0.202
Insulin-dependent DM	69 (21.8)	–	–	60 (20.8)	–	–
**Baseline medication**						
Aspirin	313 (99.1)	400 (99.3)	1.000	286 (99.0)	287 (99.3)	1.000
Clopidogrel	315 (99.7)	403 (100.0)	0.439	289 (100.0)	289 (100.0)	1.000
Ticagrelor	1(0.3)	–	–	–	–	–
Statin	305 (96.5)	390 (96.8)	0.850	279 (96.5)	279 (96.5)	1.000
β blocker	300 (94.9)	369 (91.6)	0.078	274 (94.8)	269 (93.1)	0.383
CCB	144 (45.6)	188 (46.7)	0.773	137 (47.4)	131 (45.3)	0.617

*Values are presented as mean ± standard deviation or number (%). LVEF, left ventricular ejection fraction; eGFR, estimated glomerular filtration rate; LDL-C, low-density lipoprotein cholesterol; PCI, percutaneous coronary intervention; MI, myocardial infarction; CABG, coronary artery bypass grafting; CAD, coronary artery disease; COPD, chronic obstructive pulmonary disease; PVD, peripheral vessel disease; DM, diabetes mellitus; CCB, calcium channel blocker*.

**Table 2:** Lesion and treatment characteristics in the diabetes and the non-diabetes groups.

**Variables**	**Total population (** ***n*** **=** **719)**		***P*-value**	**Propensity-matched patients (** ***n*** **=** **578)**		***P*-value**
	**Diabetes (*n* = 316)**	**Non-diabetes (*n* = 403)**		**Diabetes (*n* = 289)**	**Non-diabetes (*n* = 289)**	
**Characteristics of CTO lesion**						
One CTO lesion	244 (77.2)	301 (74.7)	0.433	220 (76.1)	216 (74.7)	0.699
Two CTO lesions	41 (13.0)	68 (16.9)	0.148	39 (13.5)	48 (16.6)	0.295
Location of CTO lesions						
LAD	131 (41.5)	163 (40.4)	0.785	116 (40.1)	118 (40.8)	0.865
LCX	57 (18.0)	57 (14.1)	0.156	54 (18.7)	41 (14.2)	0.145
RCA	132 (41.8)	186 (46.2)	0.240	123 (42.6)	133 (46.0)	0.402
Multivessel disease	267 (84.5)	331 (82.1)	0.401	245 (84.8)	236 (81.7)	0.316
Proximal or mid	240 (75.9)	324 (80.4)	0.150	219 (75.8)	233 (80.6)	0.158
Severe Calcification	20 (6.3)	23 (5.7)	0.727	19 (6.6)	22 (7.6)	0.627
Length ≥ 20 mm	283 (89.6)	373 (92.6)	0.158	258 (89.3)	268 (92.7)	0.146
Angulation > 45°	59 (18.7)	61 (15.1)	0.207	59 (20.4)	69 (23.9)	0.316
Vessel diameter (mm)	2.97 ± 0.5	2.99 ± 0.5	0.397	2.97 ± 0.5	2.99 ± 0.5	0.621
SYNTAX score	17.30 ± 9.0	17.30 ± 8.6	0.997	17.26 ± 9.1	17.17 ± 9.0	0.909
J-CTO score	1.17 ± 0.6	1.16 ± 0.5	0.877	1.16 ± 0.59	1.24 ± 0.57	0.100
**Treatment characteristics**						
Number of stents for CTO-PCI						
1	48 (15.2)	75 (18.6)	0.227	43 (14.9)	52 (18.0)	0.312
2	110 (34.9)	144 (35.7)	0.821	100 (34.6)	100 (34.6)	1.000
≥3	109 (34.5)	140 (34.7)	0.945	101 (34.9)	103 (35.6)	0.862
Stent length (mm)	53.42 ± 26.4	54.09 ± 25.5	0.742	52.98 ± 26.3	55.44 ± 26.4	0.283
IVUS use	38 (12.0)	52 (12.9)	0.724	35 (12.1)	38 (13.1)	0.707

*Values are presented as mean ± standard deviation or number (%). CTO, chronic total occlusion; LAD, left ascending coronary artery; LCX, left circumflex coronary artery; RCA, right coronary artery; J-CTO, Japanese-chronic total occlusion; PCI, percutaneous coronary intervention; IVUS, intravenous ultrasound*.

### Follow-Up Outcomes

**Figure 2:**
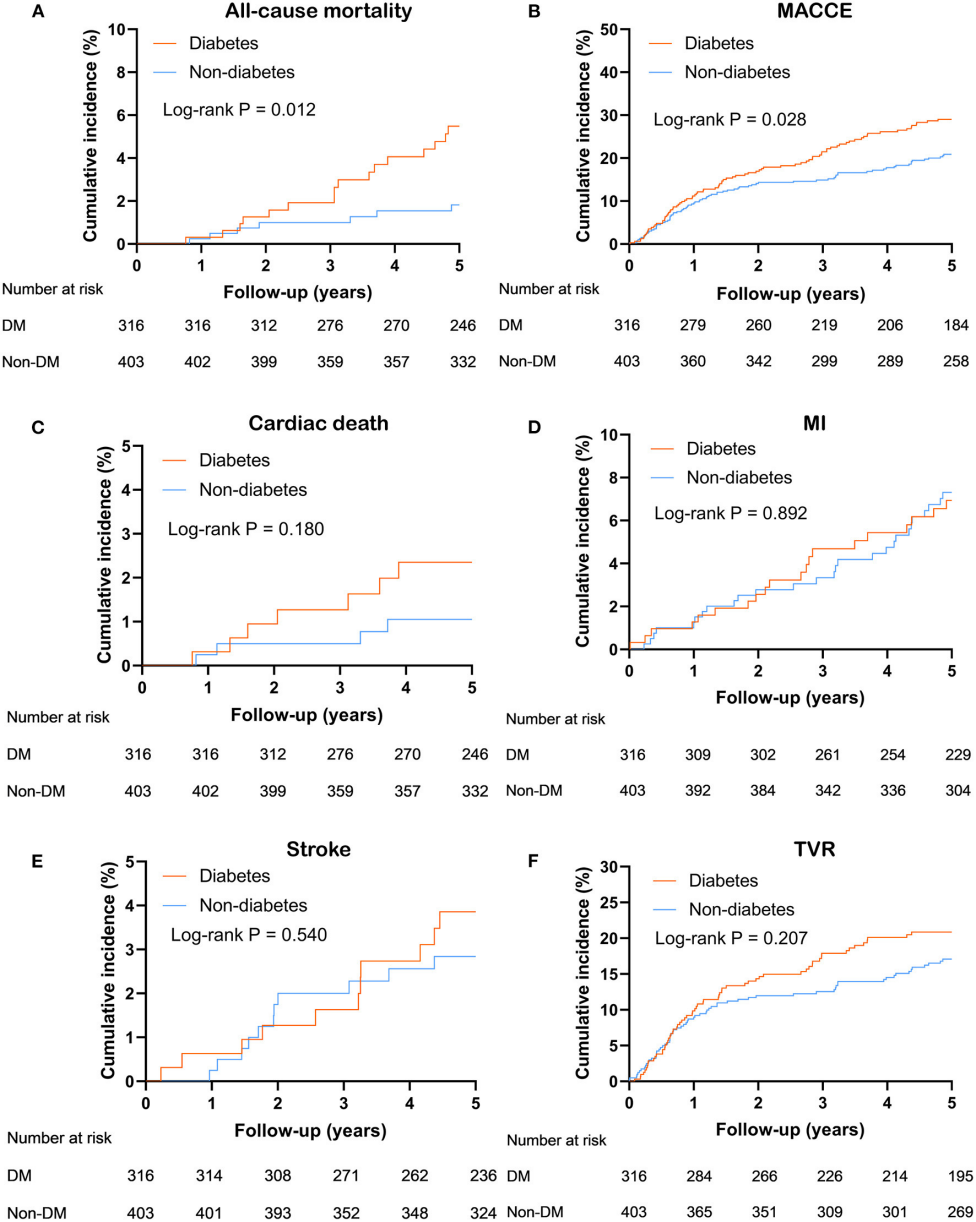
Kaplan Meier survival curves for 5 years **(A)** all-cause mortality; **(B)** MACCE; **(C)** cardiac death; **(D)** MI; **(E)** stroke; **(F)** TVR in entire population. MACCE, major adverse cardiac and cerebrovascular events; MI, myocardial infarction; TVR, target-vessel revascularization.

Over a median follow-up time was 5 (interquartile range: 2.5–5.1) years, 23 (3.2%) deaths and 175 (24.3%) MACCE occurred. DM group had a higher incidence of MACCE (diabetes vs. non-diabetes: 28.5 vs. 21.1%, unadjusted hazard ratio [HR] 1.40, 95% confidence interval [CI] 1.04–1.88, *P* = 0.028) and all-cause mortality (diabetes vs. non-diabetes: 5.1 vs. 1.7%, adjusted HR 2.97, 95% CI 1.22–7.23, *P* = 0.016). Kaplan-Meier curve analysis showed that similar results (Figure 2). Through multivariate analysis, we found that the MACCE risk was significantly higher in the diabetic patients compared to the non-diabetic patients (adjusted HR 1.47, 95% CI 1.08–2.00, *P* = 0.013). However, the occurrence of all-cause mortality (adjusted HR 2.37, 95% CI 0.94–5.98, *P* = 0.068) was not significantly different between the diabetic and non-diabetic groups (Table 3).

**Table 3:** Risk of various clinical outcomes up to 5 years in all patients.

**Outcomes**	**Incidence of event at 5 years [** ***n*** **(%)]**		**Crude HR (95% CI)**	***P*-value**	**Adjusted HR (95% CI)**	***P*-value**
	**Diabetes (*n* = 316)**	**Non-diabetes (*n* = 403)**				
All-cause mortality	16 (5.1)	7 (1.7)	2.97 (1.22–7.23)	0.016	2.37 (0.94–5.98)	0.068
Cardiac death	7 (2.2)	4 (1.0)	2.26 (0.66–7.73)	0.192	1.17 (0.30–4.60)	0.822
MI	23 (7.3)	32 (7.9)	0.93 (0.54–1.59)	0.790	0.91 (0.52–1.59)	0.744
Stroke	11 (3.5)	11 (2.7)	1.30 (0.56–2.99)	0.541	1.00 (0.43–2.35)	1.000
TVR	64 (20.3)	67 (16.6)	1.25 (0.89–1.76)	0.204	1.28 (0.90–1.81)	0.169
MACCE	90 (28.5)	85 (21.1)	1.40 (1.04–1.88)	0.028	1.47 (1.08–2.00)	0.013

*MI, myocardial infarction; TVR, target-vessel revascularization; MACCE, major adverse cardiac and cerebrovascular events*.

In propensity score-matched patients, Cox regression analyses showed no significant differences between the two matched groups with regards to the prevalence of MACCE (diabetes vs. non-diabetes: 29.1 vs. 23.2%, unadjusted HR 1.27, 95% CI 0.92–1.76, *P* = 0.141) and all-cause mortality (diabetes vs. non-diabetes: 4.5 vs. 1.7%, unadjusted HR 2.66, 95% CI 0.95–7.47, *P* = 0.063). The results of univariable and multivariable analyses showed that the risk for the primary and secondary clinical outcomes was similar between the two matched group after PSM (Table 4).

**Table 4:** Risk of various clinical outcomes up to 5 years in propensity-matched patients.

**Outcomes**	**Incidence of event at 5 years [** ***n*** **(%)]**		**Crude HR (95% CI)**	***P*-value**	**Adjusted HR (95% CI)**	***P*-value**
	**Diabetes (*n* = 316)**	**Non-diabetes (*n* = 403)**				
All-cause mortality	13 (4.5)	5 (1.7)	2.66 (0.95–7.47)	0.063	2.56 (0.91–7.24)	0.076
Cardiac death	4 (1.4)	3 (1.0)	1.36 (0.30–6.06)	0.690	1.18 (0.25–5.50)	0.835
MI	22 (7.6)	24 (8.3)	0.93 (0.52–1.66)	0.807	0.94 (0.53–1.68)	0.835
Stroke	11 (3.8)	6 (2.1)	1.88 (0.69–5.07)	0.216	1.00 (0.39–2.60)	1.000
TVR	61 (21.1)	54 (18.7)	1.13 (0.79–1.64)	0.502	1.13 (0.78–1.64)	0.509
MACCE	84 (29.1)	67 (23.2)	1.27 (0.92–1.76)	0.141	1.27 (0.92–1.75)	0.155

*MI, myocardial infarction; TVR, target-vessel revascularization; MACCE, major adverse cardiac and cerebrovascular events*.

Additionally, after adjustment of underlying confounding factors using the same method of previous Cox regression analysis, we did not find significant difference between the two groups in the risk of MACCE (adjusted HR 1.37, 95% CI 0.93–2.03, *P* = 0.106) and all-cause mortality (adjusted HR 1.14, 95% CI 0.28–4.63, *P* = 0.849) at 2 years (Details available in Supplementary Table 2).

**Figure 3:**
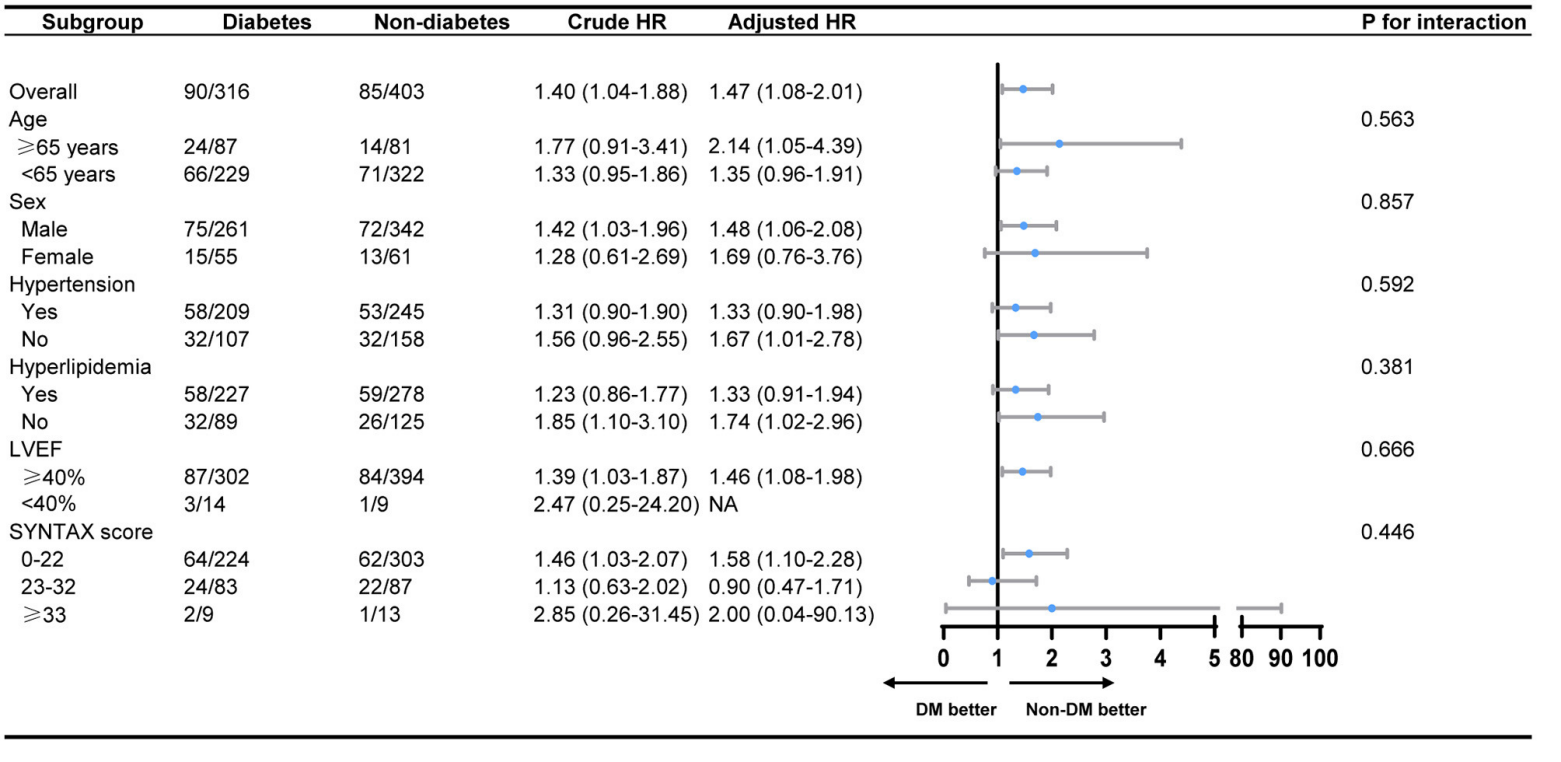
Subgroup analysis on MACCE between the diabetes group and the non-diabetes group. MACCE, major adverse cardiac and cerebrovascular events; LVEF, left ventricular ejection fraction; LAD, left ascending coronary artery; NA, not applicable.

*Post-hoc* subgroup analysis showed no significant interactions following MACCE between those covariates (age, sex, hypertension, hyperlipidemia, LVEF and SYNTAX score, all *P* for interaction > 0.05) and patients' glycemic status (Figure 3). In diabetic patients with successful CTO-PCI, stratification analysis further showed that patients in the less-controlled DM group were not at higher risk of MACCE, compared with patients in the controlled DM. Similar result was also found between insulin-dependent DM and non-insulin-dependent DM (Table 5).

**Table 5:** Stratification analysis on 5-year MACCE in the diabetes group.

**Stratification**	**Adjusted HR (95% CI)**	***P-*value**
Less-controlled DM (*n* = 169)	1.05 (0.68–1.62)	0.822
Controlled DM (*n* = 147)		
Insulin-dependent DM (*n* = 69)	1.34 (0.82–2.20)	0.241
Non-insulin-dependent DM (*n* = 247)		

## Discussion

We assessed the 5-year cardiovascular survival of successful CTO-PCI patients with or without DM in a prospective and real-world cohort population. Notably, we confirmed the following: (1) Non-diabetic patients were related to better long-term survival benefit in terms of MACCE for the treatment of successful CTO-PCI. (2) The risk of 5-year MACCE appeared to be comparable in less-controlled and controlled diabetic patients after successful recanalization of CTO.

With substantial and significant improvement in interventional devices and techniques, CTO-PCI has emerged as an effective revascularization strategy with high success rates for diabetic patients. Moreover, it is well-established that DM represents an important risk equivalent of CTO and an independent factor for increased MACE after CTO-PCI (23, 24). Sanguineti et al. reported that DM was a significant predictor of cardiac mortality in CTO patients (25). Additionally, Yan et al. found that both successful CTO-PCI and CTO-CABG of right coronary artery in diabetic patients showed significant reduction of all-cause death (HR 0.445, 95% CI 0.278–0.714) during long-term follow-up (26). Recently, Guo et.al also reported that in DM group, successful CTO-PCI reduced MACE risk (HR 0.61, 95% CI 0.42–0.87, *P* = 0.005) compared to optimal medical therapy alone (27). Likewise, Tsai et al. also found that DM was associated with poor prognosis in patients with CTO lesions compared with non-DM (14). Moreover, this study also showed that successful CTO-PCI was independently associated with reduced risks of all-cause death and adverse cardiovascular events only in DM population, but not in non-DM patients, which was consistent with the finding of Guo and co-workers (27). These evidences highlighted the unfavorable role of DM in CTO patients and the importance of complete recanalization of CTO patients with DM. Contrary to the results of previous findings, subgroup analysis of the randomized COURAGE trial demonstrated that there was no obvious difference in the incidence of adverse events between the medical therapy group and the PCI group in DM patients with stable coronary disease (28). This difference may be explained by the high rate (~30%) of crossover from medication to revascularization during the follow-up period, which may underestimate the actual effect of successful CTO-PCI.

Considerable evidence has demonstrated that the existence of DM has a detrimental effect on glucose and lipid metabolism, endothelial function and angiogenesis, leading to premature development and progression of coronary artery atherosclerosis, inadequate collateral development and harmful clinical outcomes (29–31). Previous studies have showed that well-established collateral circulation after CTO is crucial to supply the downstream perfusion area, alleviate myocardial damage, reduce infarct size, improve LVEF and eventually decrease adverse events (32, 33). This may explain the worse prognosis on diabetic patients with successful CTO-PCI. However, recently, Yang et al. reported that after successful recanalization of CTO, there was no significant distinction between diabetic and non-diabetic effects of coronary collaterals on MACCE and repeat revascularization during a median follow-up of 13.5 months (34). Yang and co-workers speculated that well-developed coronary collaterals may not adequately substitute normal blood supply and thus good collateral circulation is insufficient.

Recently, with regard to the long-term clinical outcomes of successful CTO-PCI in patients with vs. without DM, a meta-analysis by Zhu et al. which included 9,847 patients after successful CTO-PCI (4,238 diabetic patients and 5,069 non-diabetic patients) revealed that the prevalence of MACEs (RR 1.26, 95% CI 1.02–1.56, *P* = 0.03) was significantly higher, compared with patients without DM (35). Likewise, consistent with Guo and co-workers (27), our study also reported that the rates of MACCE after successful CTO-PCI were higher in diabetic patients than in non-diabetic patients. In contrast, Ruiz Garcia et al. reported that in patients who underwent successful revascularization of CTO comparable rate of MACE was observed between the diabetic and non-diabetic patients in the drug-eluting stent era (36). Although this was a prospective randomized clinical study, the atypical definition of CTO (occlusion longer than 2 weeks), the small sample size of its enrolled patients (75 diabetic and 132 non-diabetic patients) and the modest follow-up period of 1 year restricted the accuracy of the results. In our study, we also found that the prevalence of 2-year (shorter term) clinical outcomes was comparable between the diabetic patients and non-diabetic patients, which was consistent with the findings of Ruiz Garcia and co-workers. Thus, it is necessary to evaluate longer term prognosis for diabetic patients undergoing successful CTO-PCI.

Besides, we found that diabetic patients with less-controlled DM were not at a higher risk of 5-year MACCE, compared with those with controlled DM, which was consistent with the findings of the randomized VADT trial (37). It demonstrated that intensive glucose control had shown no evidence of cardiovascular or overall survival benefit during the median follow-up of 5.6 years. However, Holman et al. reported that after longer-term (about 10 years) observational follow-up, both in the sulfonylurea-insulin group and the metformin group, diabetic patients with glycemic control had significant reductions in MI and all-cause mortality (38). We speculated that the reasons for the inconsistent findings of previous studies may be the different population characteristics and therapeutic approaches. Further randomized controlled trials with longer term follow-up are warranted to validate our results.

Our study had some inevitable limitations. First, it was a single-center, prospective and observational study. Although we performed propensity score matching to reduce potential selection bias and minimize the confounding factors, unadjusted confounders still existed. Second, our real-world study is a *post-hoc* analysis of a consecutively enrolled cohort of CAD patients undergoing PCI. Since this was not a dedicated CTO cohort, we expected the sample size of CTO patients to be modest when designing the study. Third, there was a lack of specific information in our database, such as coronary collateral scoring and the glycemic control during the long follow up, which may impair the precise evaluation of future risk of adverse events in CTO patients. Fourth, our center was a tertiary medical hospital which performed high volume of CTO-PCI and had many experienced cardiologists. Generalizability might be limited in less experienced center with lower number of CTO-PCI cases. In fact, previous studies have indicated that patients in DM group were more likely to have complex clinical characteristics (9, 13). However, in our study, baseline clinical and lesion characteristics were comparable between the diabetic and non-diabetic groups, which may be partially interpreted by these limitations above.

## Conclusions

The present study suggests that diabetic patients with successful CTO-PCI encountered more long-term adverse clinical outcomes, based on their complex lesions and co-morbidities. After a successful CTO-PCI, non-diabetic patients were associated with better long-term survival benefit in terms of MACCE. The risk of 5-year MACCE appeared to be comparable in less-controlled and controlled diabetic patients. These findings may provide clinical insight into treatment option for unselected patients with diabetes. Further randomized controlled trials with longer term follow-up are required to validate our results.

## Data Availability Statement

The raw data supporting the conclusions of this article will be made available by the authors, without undue reservation.

## Ethics Statement

The studies involving human participants were reviewed and approved by The Institutional Ethics Committee at Fu Wai Hospital. The patients/participants provided their written informed consent to participate in this study. Written informed consent was obtained from the individual(s) for the publication of any potentially identifiable images or data included in this article.

## Author Contributions

PW, DY, SJ, RG, and JY contributed to the study design and interpretation of the results. PZ, LJ, YS, JX, XT, CZ, SJ, YL, DY, and TL contributed to the collection, analysis, or interpretation of data. PW prepared the manuscript. JY, RG, BX, YY, XZ, SJ, and DY critically revised the manuscript. All authors read and approved the final submitted version.

## Conflict of Interest

The authors declare that the research was conducted in the absence of any commercial or financial relationships that could be construed as a potential conflict of interest.

## Publisher's Note

All claims expressed in this article are solely those of the authors and do not necessarily represent those of their affiliated organizations, or those of the publisher, the editors and the reviewers. Any product that may be evaluated in this article, or claim that may be made by its manufacturer, is not guaranteed or endorsed by the publisher.
