# Supplementary material for: 5-Year Clinical Outcomes of Successful Recanalisation for Coronary Chronic Total Occlusions in Patients With or Without Type 2 Diabetes Mellitus
Source: Front Cardiovasc Med. 2021 Aug 13;8:691641. doi: 10.3389/fcvm.2021.691641 (PMC8414521; doi:10.3389/fcvm.2021.691641)
Supplement: Supplementary file 1 [file Data_Sheet_1.PDF]

Table S1 Univariable and multivariable analysis of successful revascularization on 5-year endpoints before matching

Variable	All cause mortality				MACCE			
	univariable analysis		multivariable analysis		univariable analysis		multivariable analysis	
	HR	p value	HR	p value	HR	p value	HR	p value
Age	1.042<br>1.000-1.085	0.050	1.031<br>0.975-1.091	0.282	0.998<br>0.984-1.013	0.807	1.011<br>0.991-1.030	0.280
Sex	1.114<br>0.379-3.276	0.844			0.991<br>0.661-1.484	0.965		
Current smoking	1.123<br>0.476-2.648	0.792			0.918<br>0.677-1.244	0.581		
Hypertension	1.340<br>0.551-3.258	0.518			1.002<br>0.736-1.362	0.992		
Hypercholesterolemia	0.785<br>0.333-1.851	0.785			0.812<br>0.593-1.113	0.196		
eGFR	0.968<br>0.947-0.990	0.004	0.988<br>0.957-1.021	0.481	1.004<br>0.994-1.015	0.404	1.014<br>1.001-1.028	0.038
LVEF (%),at baseline	0.947<br>0.905-0.992	0.020	0.976<br>0.926-1.028	0.357	0.991<br>0.973-1.010	0.361	0.996<br>0.975-1.017	0.677
Prior stroke	3.614<br>0.487-26.812	0.209	1.880<br>0.184-19.184	0.594	8.648<br>4.367-17.127	0.000	9.730<br>4.666-20.289	0.000
Prior PCI	1.806<br>0.766-4.259	0.177	1.482<br>0.598-3.672	0.395	1.466<br>1.058-2.031	0.021	1.387<br>0.987-1.949	0.059
Prior MI	2.660<br>1.174-6.029	0.019	1.821<br>0.721-4.597	0.205	1.231<br>0.898-1.686	0.196	1.010<br>0.709-1.437	0.957
LAD involvement	0.783	0.577	0.859	0.739	0.742	0.061	0.734	0.056

	0.332-1.848		0.353-2.094		0.543-1.014		0.535-1.007	
PVD	3.820<br>0.896-16.292	0.070	3.211<br>0.625-16.490	0.162	2.008<br>1.027-4.245	0.042	1.919<br>0.919-4.009	0.083
Multivessel disease	1.318<br>0.392-4.436	0.655			1.296<br>0.842-1.994	0.238		
J-CTO score	1.024<br>0.490-2.141	0.950			1.090<br>0.834-1.424	0.527		
SYNTAX score	0.978<br>0.934-1.025	0.356			0.991<br>0.975-1.008	0.310		

eGFR: estimated glomerular filtration rate; LVEF: left ventricular ejection fraction; PCI: percutaneous coronary intervention; MI: myocardial infarction; LAD: left ascending coronary artery; PVD: peripheral vessel disease; J-CTO: Japanese-chronic total occlusion.

Table S2 Risk of various clinical outcomes up to 2 years in all patients

Outcomes	Incidence of event at 2 years [n (%)]		Crude HR (95% CI)	P value	Adjusted HR (95% CI)	P value
	Diabetes (n=316)	Non-diabetes (n=403)				
All-cause mortality	7 (2.2)	4 (1.0)	2.21 (0.65-7.55)	0.206	1.14 (0.28-4.63)	0.849
Cardiac death	5 (1.6)	1 (0.2)	6.35 (0.74-54.34)	0.092	0.62 (0.04-8.75)	0.726
MI	5 (1.6)	11 (2.7)	0.58 (0.20-1.67)	0.313	0.51 (0.17-1.56)	0.239
Stroke	3 (0.9)	6 (1.5)	0.65 (0.16-2.58)	0.536	1.00 (0.26-3.80)	1.000
TVR	44 (13.9)	44 (10.9)	1.28 (0.84-1.94)	0.248	1.30 (0.85-1.98)	0.228
MACCE	55 (17.4)	54 (13.4)	1.31 (0.90-1.91)	0.158	1.37 (0.93-2.03)	0.106

MI: myocardial infarction; TVR: target-vessel revascularization; MACCE: major adverse cardiac and cerebrovascular events.
